# Emergence of bottom-up saliency in a spiking model of V1

**DOI:** 10.1186/1471-2202-14-S1-P183

**Published:** 2013-07-08

**Authors:** Botond Szatmáry, Micah Richer, Jayram Moorkanikara Nageswaran, Csaba Petre, Filip Piekniewski, Sach Sokol, Eugene M Izhikevich

**Affiliations:** 1 Brain Corporation, San Diego, CA 92121, USA

## 

We present anatomically detailed spiking model of the parvo and magno pathways of the retina, primary visual cortex (V1), and superior colliculus (SC) to enable active saccades. Due to STDP and visual experience, the model shows the emergence of a saliency map, resulting in the perceptual behavior of bottom-up (pop-out) attention.

In contrast to previous models proposed to explain pop-out based attention for simple features (e.g., saliency map hypothesis of [1]), where feature selectivity and inhibitory mechanisms between similar features are pre-wired, connectivity in our spiking model is neither pre-wired nor are neurons pre-labeled, but feature selectivity and pop-out behavior still emerges.

Projections between cell types in the V1 model (L4 and L2/3) are in agreement with anatomical data. Both excitatory and inhibitory synapses are subject to different forms of STDP. These plasticity mechanisms and exposure to rich natural visual stimuli lead to (i) neuronal responses similar to those recorded in vivo, (ii - parvo) formation in color selective cells, and (iii - magno) formation of simple and complex cells covering a broad range of orientations and spatial frequencies.

Pop-out mechanism is mediated by modifying the activity in layer 2/3 with long-range effective inhibition using a narrow form of STDP, which selectively picks up short temporal correlations between neurons responding to similar features but depresses ignores neurons responding to different features. Stronger within-feature long-range inhibition dampens the population response to features that are abundant in the input, but allows strong response to salient input features. The activity of V1 drives the SC, resulting in pop-out saccades. (The SC model is presented in a separate submission.)

The model connects electrophysiology (spiking activity) and perception, and it explains animal behavior in a variety of standard pop-out tasks.

Neurons responding to vertical features will receive strong inhibition from other vertical neurons, therefore weakening their response, while response triggered by the single horizontal bar remains strong.

**Figure 1:**
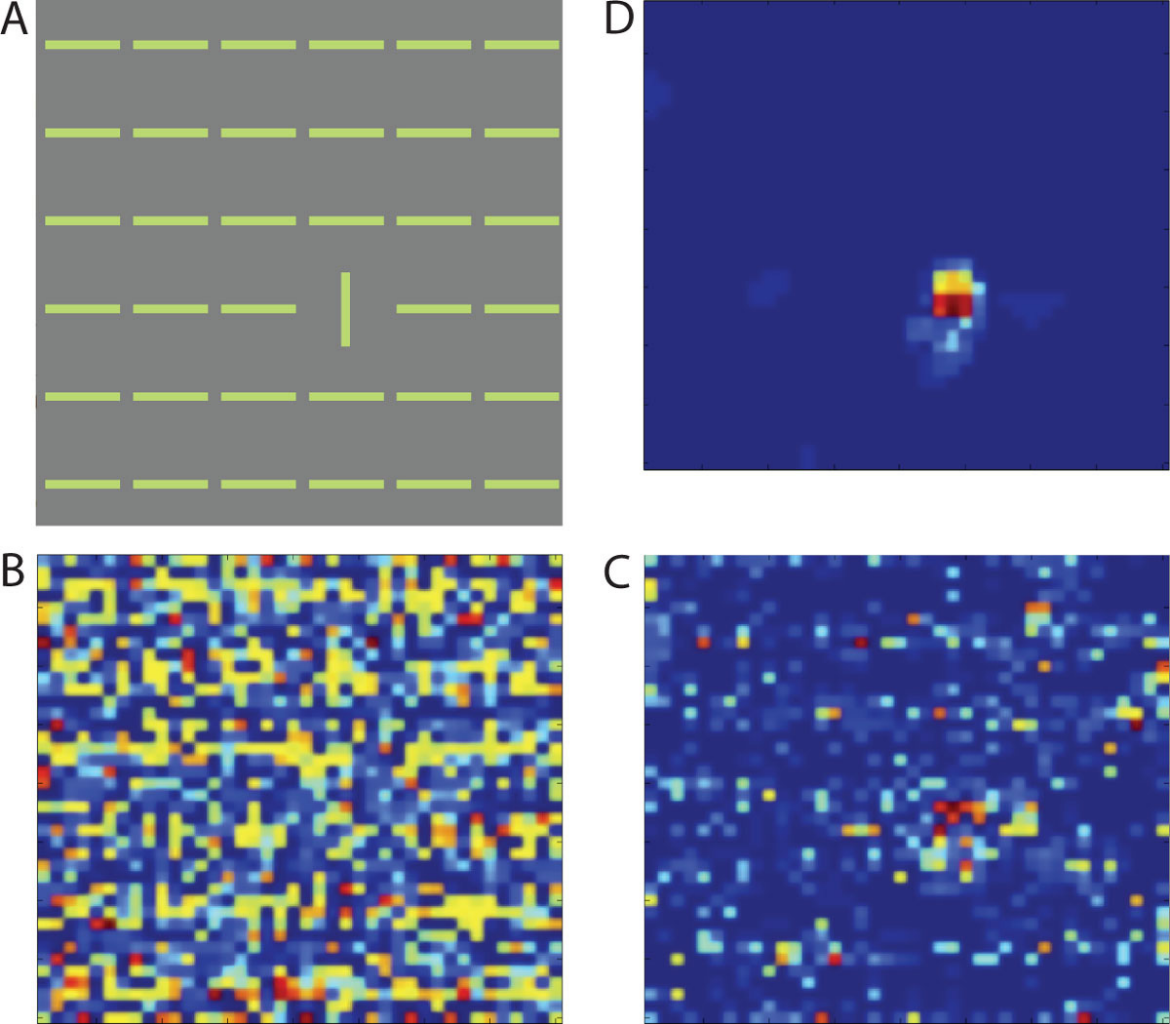
**A. Input image; B. V1 layer 2/3 activity without long-range inhibition; C. V1 layer 2/3 activity with long-range inhibition; D. Superior colliculus activity that directly drives the saccadic mechanism**. Activity is averaged over two seconds.

## References

[B1] Itti L Koch C Computational modelling of visual attention Nature Reviews Neuroscience 2001 2 3 194 203 10.1038/35058500 11256080

